# Dual microRNA Screens Reveal That the Immune-Responsive miR-181 Promotes Henipavirus Entry and Cell-Cell Fusion

**DOI:** 10.1371/journal.ppat.1005974

**Published:** 2016-10-26

**Authors:** Chwan Hong Foo, Christina L. Rootes, Karla Cowley, Glenn A. Marsh, Cathryn M. Gould, Celine Deffrasnes, Christopher J. Cowled, Reuben Klein, Sarah J. Riddell, Deborah Middleton, Kaylene J. Simpson, Lin-Fa Wang, Andrew G. D. Bean, Cameron R. Stewart

**Affiliations:** 1 CSIRO Health and Biosecurity, Australian Animal Health Laboratory, Geelong, Victoria, Australia; 2 Victorian Centre for Functional Genomics, Peter MacCallum Cancer Centre, Melbourne, Victoria, Australia; 3 The Sir Peter MacCallum Department of Oncology, University of Melbourne, Melbourne, Australia; 4 Program in Emerging Infectious Diseases, Duke-NUS Graduate Medical School, Singapore; Georgia State University, UNITED STATES

## Abstract

Hendra and Nipah viruses (family *Paramyxoviridae*, genus *Henipavirus*) are bat-borne viruses that cause fatal disease in humans and a range of other mammalian species. Gaining a deeper understanding of host pathways exploited by henipaviruses for infection may identify targets for new anti-viral therapies. Here we have performed genome-wide high-throughput agonist and antagonist screens at biosafety level 4 to identify host-encoded microRNAs (miRNAs) impacting henipavirus infection in human cells. Members of the miR-181 and miR-17~93 families strongly promoted Hendra virus infection. miR-181 also promoted Nipah virus infection, but did not affect infection by paramyxoviruses from other genera, indicating specificity in the virus-host interaction. Infection promotion was primarily mediated via the ability of miR-181 to significantly enhance henipavirus-induced membrane fusion. Cell signalling receptors of ephrins, namely EphA5 and EphA7, were identified as novel negative regulators of henipavirus fusion. The expression of these receptors, as well as EphB4, were suppressed by miR-181 overexpression, suggesting that simultaneous inhibition of several Ephs by the miRNA contributes to enhanced infection and fusion. Immune-responsive miR-181 levels was also up-regulated in the biofluids of ferrets and horses infected with Hendra virus, suggesting that the host innate immune response may promote henipavirus spread and exacerbate disease severity. This study is the first genome-wide screen of miRNAs influencing infection by a clinically significant mononegavirus and nominates select miRNAs as targets for future anti-viral therapy development.

## Author Summary

The henipaviruses Hendra and Nipah are bat-borne paramyxoviruses that are highly pathogenic in humans. Until recently the constraints of working at biosafety level 4 had hindered the large scale study of host factors associated with henipavirus infection. MicroRNAs are a class of single-stranded non-coding RNAs that regulate biological processes in eukaryotes. An emerging body of evidence suggests that host microRNAs may favour infection of vertebrate RNA viruses. We have performed high-throughput agonist and antagonist screens at biosafety level 4 to identify host-encoded microRNAs impacting henipavirus infection in human cells. Members of the miR-181 and miR-17~93 families strongly promoted Hendra virus infection and appear to suppress multiple antiviral host molecules. Infection promotion is primarily mediated via the ability of miR-181 to repress Eph receptors that negatively regulate henipavirus glycoprotein-mediated cell-cell fusion. This study is the first large-scale screen of host-encoded microRNAs influencing infection by a clinically significant mononegavirus, and of a BSL-4 virus, and supports the emerging notion that host miRNAs can play a role in supporting infection of RNA viruses.

## Introduction

Hendra virus (HeV) and Nipah virus (NiV) are highly pathogenic zoonotic paramyxoviruses belonging to the genus *Henipavirus* [1]. First isolated in Australia in 1994, HeV disease has caused seven clinically confirmed human cases with four fatalities. NiV initially appeared in Malaysia in 1998–1999, resulting in 105 human fatalities. Since 2001, recurring outbreaks of NiV have been reported in South Asia, resulting in more than 211 deaths and an average case-fatality rate of approximately 75% [2, 3]. Both bat-borne henipaviruses cause severe respiratory illness and encephalitis in humans, however there is a lack of therapies and vaccines. With high fatality rates emphasising the need for effective anti-viral strategies [4–6], a better understanding of henipavirus biology is required.

Viruses may co-opt or alter a range of host cell processes that optimise replicative efficiency. One such process is the RNA interference (RNAi) pathway [7]. Conventionally, in chordates RNAi involves the base-pairing of small non-coding microRNA (miRNA) molecules in a multi-protein complex to complementary mRNA sequences, often resulting in post-transcriptional silencing of host gene expression [8]. Some DNA viruses (i.e. herpesviruses) in particular, which also encode their own viral miRNAs, are known to subvert this fundamental host process to promote infection [7]. For RNA viruses however, the pro-viral roles of host miRNAs remain poorly characterized. Up until recently, the general thought was that the multifaceted dependence of hepatitis C virus infection on hepatocyte-specific miR-122 is the exception, not the rule, for RNA viruses [9, 10].

More recently, a few high profile studies have highlighted that the usurping of host miRNAs by RNA viruses might previously have been underappreciated. Trobaugh et al. showed that the alphavirus Eastern equine encephalitis virus (EEEV) utilizes host-derived miR-142-3p to define cell tropism and to suppress innate immunity, indirectly promoting neuropathogenesis [11]. A comprehensive survey of 15 RNA viruses from 7 families identified miR-17 and let-7 binding to pestivirus 3’ UTR as critical for enhanced viral translation, RNA stability and virus production [12]. The Argonaute protein, a key component of functional miRNA complexes, was also found to be associated with viral RNA of virtually all of the viruses assessed, including paramyxoviruses [12]. These Argonaute-viral RNA interactions also often exhibit preferential clustering on the viral subgenomes, implying specificities in the miRNA targeting. Enterovirus infection induces host miR-141 expression, which is then co-opted by the virus to silence cellular translation initiation factor eIF4E, resulting in host translational shutoff [13]. One emerging concept from such studies is the sequestration or “sponging” of anti-viral host miRNAs by genomes of some RNA viruses to derepress cellular transcripts that might enhance infection [9, 12]. These reports suggest that RNA viruses can adopt host miRNAs for their own utility via a diversity of mechanisms, and that this aspect of virus-host interactions is currently understudied.

With technological advancements in high-throughput techniques making the comprehensive study of both physical and genetic virus-host interactions a possibility [5], we have started executing functional genomics screens using fully infectious biosafety level 4 agents [14]. Despite the power of functional genomics as a research tool, thus far only two comprehensive RNAi screens investigating the contributions of miRNAs to pathogenesis of RNA viruses have been reported [15, 16]. No such study has been done for BSL-4 viruses and for any of the medically relevant mononegaviruses, such as paramyxoviruses or filoviruses. In light of recent studies underscoring the potential significance of miRNAs for RNA virus replication as well as the therapeutic promise of miRNA antagonists [6, 17], we sought to address this gap in our knowledge of virus-host interplay.

Here we present findings from two high-throughput genome-wide screens, conducted at BSL-4, of host-encoded miRNAs associated with HeV infection. The screens, in addition to subsequent validation work, demonstrate a key role for miR-181 family members in regulating henipavirus syncytia formation and infection, and suggest several host miRNAs, including miR-17~93, as potential candidates for novel therapeutic targets.

## Results

### High biocontainment genome-wide analysis of host-encoded miRNAs modulating henipavirus infection

**Fig 1:**
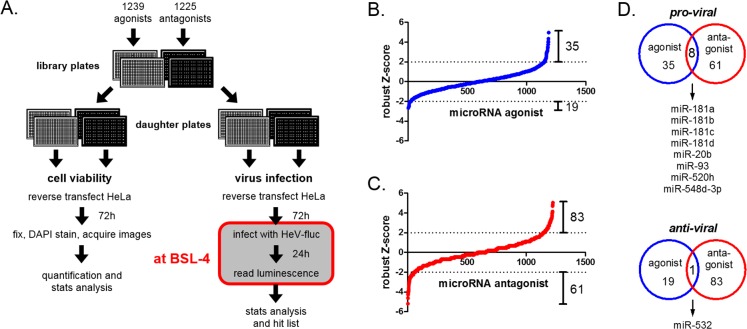
Genome-wide complementary agonist and antagonist screens of host-encoded miRNAs impacting henipavirus infection at BSL-4. (A) Schematic of optimized protocol for performing functional RNAi screens with an infectious BSL-4 virus. Two sets of daughter plates were generated from library plates consisting of 1,239 miRNA agonists (grey plates) and 1,225 antagonists (black plates). HeLa cells were added to both plate sets for reverse transfection of the miRNA agonists and antagonists. 72 h post transfection, one set of plates were processed at BSL-2 for cell viability analysis by DAPI staining. The other set was then transferred into BSL-4 (red box), infected with luciferase-expressing HeV, and lysed for luminescence reading at 24 hours post-infection (h.p.i.) in BSL-4. (B and C) Results from the miRNA agonist (B) and antagonist (C) screens, with miRNAs ranked using a robust Z-score approach, from lowest (decreased virus infection) to highest (increased virus infection). Dotted horizontal lines represent the threshold of hit identification (Z ≥ 2 or ≤ -2). The number of miRNA hits above this threshold is shown. (D) Venn diagram identifying pro- and anti-viral miRNAs from both screens.

To identify host-encoded miRNAs that regulate HeV infection, we performed two complementary high-throughput screens at BSL-4 that targeted 834 human host-encoded miRNAs (Fig 1A). This first involved the reverse transfection of HeLa cells with a library of 1,239 synthetic miRNA agonists (i.e. mimics), which are double-stranded RNA molecules that functionally imitate native miRNAs, to over-express each of 834 miRNAs in a 384-well plate format. In a concurrent screen silencing each of the 834 miRNAs, HeLa cells were transfected with a library consisting of 1,225 antagonists (i.e. inhibitors), which are single-stranded RNA molecules that bind and sequester native mature miRNAs [18]. After library preparation and transfection were performed under BSL-2 conditions, one set of the daughter plates for each screen was moved to BSL-4 (red box, Fig 1A). 72 h after transfection, these cells were infected with a recombinant HeV that expresses the firefly luciferase reporter gene [19]. After 24 h, a luminometer was used to measure luciferase expression of the infected cells for both screens at BSL-4. The impact that each miRNA agonist or antagonist had on reporter expression was normalized to values from mock-transfected cells, and then expressed in terms of a robust Z-score, which is a commonly used measure of hit identification for RNAi screens [20, 21]. Z-scores of 2 or greater indicate an increase in infection relative to control, whereas Z-scores of -2 or lower indicate a decrease in infection.

The other set of daughter plates with transfected cells were processed for cell viability analysis by nuclei quantification using an automated fluorescence microscope (Fig 1A). Cells that were transfected with a siRNA targeting the PLK1 gene served as a positive control for cell death. 54 of the agonists and none of the antagonists caused significant cell death relative to mock-transfected cells (≥70% cut-off) and were thus subsequently eliminated from the robust Z-score analysis and final hit list generation (S1 Table and S2 Table).

**Fig 2:**
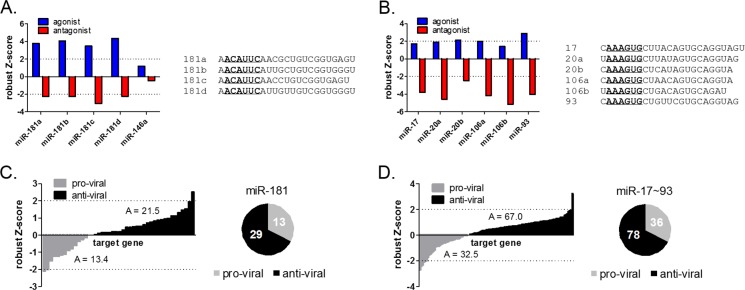
Dual miRNA screens reveal miR-181 and miR-17~93 families as promoters of henipavirus infection that target multiple anti-viral genes. (A and B) Results from miRNA screens for all miR-181 (A) and miR-17~93 (B) family members, represented by robust Z-scores. Dotted horizontal lines represent the threshold of hit identification. Mature sequences of individual miRNAs of the two families are listed, with seed regions underlined in bold. (C and D) Cross-reference analysis of verified miR-181 (C) and miR-17~93 (D) target genes with published HeV siRNA screen data [14] reveal that more than two-thirds of target genes inhibit HeV infection. Graphs on the left hand side show Z-scores of experimentally verified miRNA target genes from a published genome-wide siRNA screen of host genes impacting HeV infection. Genes with Z-scores < 0 were designated pro-viral, while genes with Z-scores > 0 were designated anti-viral. Pie charts show the relative proportions of pro- and anti-viral target genes for miR-181 (C) and for miR-17~93 (D), with the number of genes printed. Values represent the sum of all the Z-scores, and demonstrate the predominance of anti-viral genes among the miR-181 and miR-17 targets.

The agonist and antagonist screens identified 35 and 61 miRNAs respectively that significantly promoted HeV infection, and 19 and 83 miRNAs respectively, that significantly inhibited virus infection (Fig 1B and 1C, S1 Table and S2 Table). Eight miRNAs exhibited pro-viral characteristics in both agonist and antagonist screens (Fig 1D), including all four members of the miR-181 family. Conversely, both screens identified miR-532 as a miRNA that inhibits infection. In regards to miRNAs that promote virus infection, screen results from two miRNA families were notable. All four members of the miR-181 family significantly promoted HeV infection (Fig 2A). These miRNAs all share the same seed sequence (ACAUUC), implying significant congruency in function(s). The scale of the pro-viral impacts of miR-181 members is especially remarkable if we compare their effects to that of miR-146a (Fig 2A), which we previously validated as pro-viral for HeV [22]. In addition to miR-181, most members of the miR-17~93 family were pro-viral (Fig 2B). The seed sequence of the miRNAs in this family (AAAGUG) is distinct from that of miR-181.

### Verified miR-181 and miR-17~93 target genes predominantly inhibit henipavirus infection

MiRNAs regulate gene expression by binding to complementary sequences typically located in the 3’ untranslated region (3’ UTR) of the mRNA target [23–26]. Depending on the degree of complementarity, this generally results in the suppression or degradation of target mRNA, thereby preventing encoded proteins from being translated [23, 25, 26]. As each miRNA can act as a suppressor of many target genes, we hypothesized that miR-181 and miR-17~93 families promoted henipavirus infection by suppressing multiple anti-viral host genes. To test this hypothesis, we firstly mined the miRTarbase database [27] to identify all experimentally-validated target genes for miR-181 and miR17~93 families. Next, the effects of these genes on HeV infection, as represented in robust Z-scores, were cross-referenced from results of our published genome-wide siRNA screen that identified pro- and anti-viral host genes for henipaviruses [14] (S3 Table). This analysis demonstrates that for both miRNA families these genes were more likely to be anti-viral (Fig 2C and 2D). For instance, the ratio of anti-viral to pro-viral hits for validated miR-181 targets was 2.2 to 1 (Fig 2C). In contrast, this ratio for all unbiased gene hits in the entire screen was 1 to 1 [14]. Collectively, these data suggest that the net outcome of miR-181 or miR17~93 expression is a cellular microenvironment that is more conducive for henipavirus infection. The results also indicate a reasonable level of congruency between our miRNA and siRNA gene screen datasets.

We also sought to determine whether miR-181 preferentially regulates the expression of host proteins localized in a particular subcellular compartment. To this end, the list of experimentally-validated miR-181 targets (n = 78 genes) was obtained from miRTarBase was subjected to annotation enrichment analysis using the DAVID web service. Functional annotation clustering analysis was performed using default settings (Fisher’s exact test to calculate p-values, followed by multiple testing correction using the Benjamini method). This analysis demonstrated an enrichment of miR-181 target genes associated with the nucleoplasm (*p* = 4.6e-5), while proteins associated with plasma membrane localization were not significantly enriched (*p* = 0.7).

### miR-181 promotes Hendra virus infection of human cells

All four members of the miR-181 family exhibited consistent pro-viral phenotypes in both the agonist and antagonist screens (Figs 1D and 2A). We thus decided to investigate the role of this miRNA family in henipavirus infection further. Firstly, since the screens were performed using a recombinant reporter HeV, we validated the observations using a wild-type virus. Screen results suggested that miR-181d is one of the most pro-viral members of the family (Fig 2A, S1 Table and S2 Table), hence miR-181d was chosen as a representative member of the miR-181 family in the majority of subsequent experiments.

**Fig 3:**
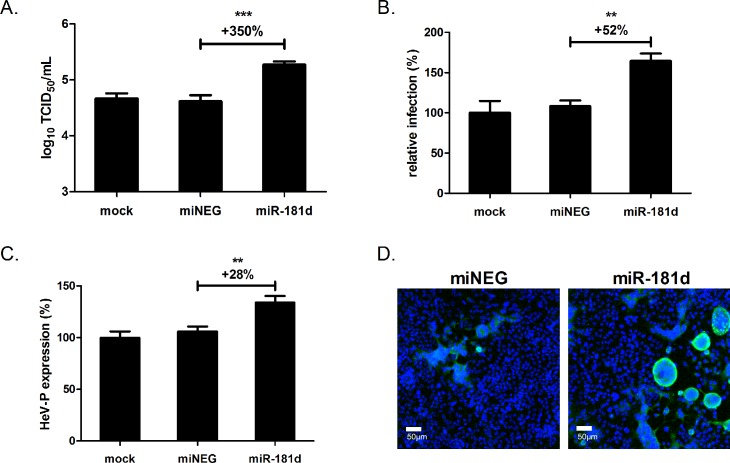
miR-181d promotes wild-type HeV infection. (A) HeV titres of supernatants from HeLa cells infected with HeV (MOI 1) for 24 h, 72 h post-transfection with miRNA agonists. (B) Percentage of the HeLa cells infected with HeV as in (A), and (C) corresponding virus P-antigen levels per infected cell, were quantitated by automated immunofluorescence imaging. (D) Immunofluorescence microscopy images showing HeLa cells treated as in (A) and stained with phosphoprotein-specific (HeV) antibody (green) and DAPI nuclear stain (blue). **p ≤ 0.01, ***p ≤ 0.001.

HeLa cells were transfected with miR-181d-specific agonists to effectively over-express miR-181d. Non-targeting miRNA agonists (miNEG), or transfection reagent devoid of agonist (mock), were also included as negative controls. At 72 h post-transfection, cells were infected with wild-type HeV and incubated for 24 h. The cell supernatants were then collected and applied to a TCID_50_ assay to quantify infectious virus titre. As indicated in Fig 3A, the results showed a 350% increase in HeV infectious titres in cells transfected with miR-181d agonists compared to control miRNA agonists.

In addition to TCID_50_ measurements of HeV titres in cell supernatants, the impact of miR-181d agonists on the proportion of infected cells and HeV protein production was investigated using quantitative immunofluorescence imaging. Transfected cells were infected with HeV for 24 h, before being fixed and stained with fluorescently labelled antibodies. The HeV phosphoprotein (P) was selected for analysis due to its high protein expression levels during infection [28, 29]. Compared with control miRNA agonists, cells transfected with miR-181d agonists showed a significant increase in both the percentage of cells infected (>50% increase) (Fig 3B) and also the levels of virus antigen per infected cell (>25% increase) (Fig 3C). In support of the quantitative data, both an increase in HeV P protein concentration and also syncytia formation (characteristic of paramyxovirus infection) could be visually observed by fluorescence microscopy (Fig 3D).

### miR-181 impacts henipavirus infection but not paramyxoviruses from other genera

**Fig 4:**
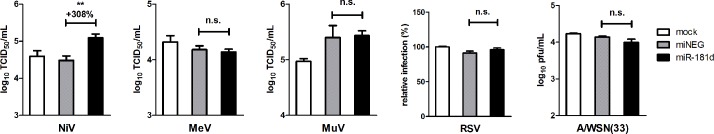
miR-181d promotes henipavirus infection specifically. Infectivity assays were applied to assess changes in virus production or virus infection of HeLa cells infected with NiV, MeV, MuV, RSV or influenza A/WSN/33 virus for 24 h. Cells were previously transfected with miR-181d or negative control agonists (miNEG) for 72 h. **p≤0.01; n.s. not significant.

To assess whether the pro-viral effects of miR-181 are specific to HeV, the *in vitro* activity of miR-181d agonists were tested on a range of viruses from different subfamilies of the *Paramyxoviridae* family. These included the closely related Nipah virus (subfamily *Paramyxovirinae*, genus *Henipavirus*), but also measles virus (subfamily *Paramyxovirinae*, genus *Morbillivirus*), mumps virus (subfamily *Paramyxovirinae*, genus *Rubulavirus*) and respiratory syncytial virus (RSV, subfamily *Pneumovirinae*, genus *Pneumovirus*). Influenza A/WSN/33(H1N1), an orthomyxovirus, was also included to compare with a virus from a different family. TCID_50_ measurements of the supernatants collected from transfected cells infected with NiV revealed a significant >300% increase in virus production in cells treated with miR-181d agonists compared to control miRNA agonists (Fig 4). On the other hand, infectivity assays showed no significant differences in virus titre between cells transfected with miR-181d or control agonists and infected with either measles virus, mumps virus, RSV or influenza A/WSN/33 (H1N1). These results indicate that the enhancement effects of miR-181 are specific to the henipavirus genus.

### Viral RNA synthesis is augmented by miR-181 over-expression

In order to narrow down the possible mechanisms by which miR-181 promotes henipavirus infection, we next sought to delineate the part of the virus life cycle at which miR-181 promotes infection. We first looked at whether viral RNA synthesis was induced by miR-181 during a single round of HeV infection. Cells were transfected with miR-181 agonists, and then infected with a high MOI (5) of HeV. At 0, 12 and 24 h post-infection (h.p.i.), cell lysates were harvested, and intracellular viral RNA levels were assessed by quantitative RT-PCR. Our previous studies have shown that in HeLa cells the first cycle of HeV infection is completed by 24 h but not at 12 h [14]. Nonetheless, here cell supernatants were also collected and virus titres from all time-points were determined by TCID_50_ assay (S1 Fig). Congruent to our previous observations, production of progeny virions was only detected at 24 h.p.i.

**Fig 5:**
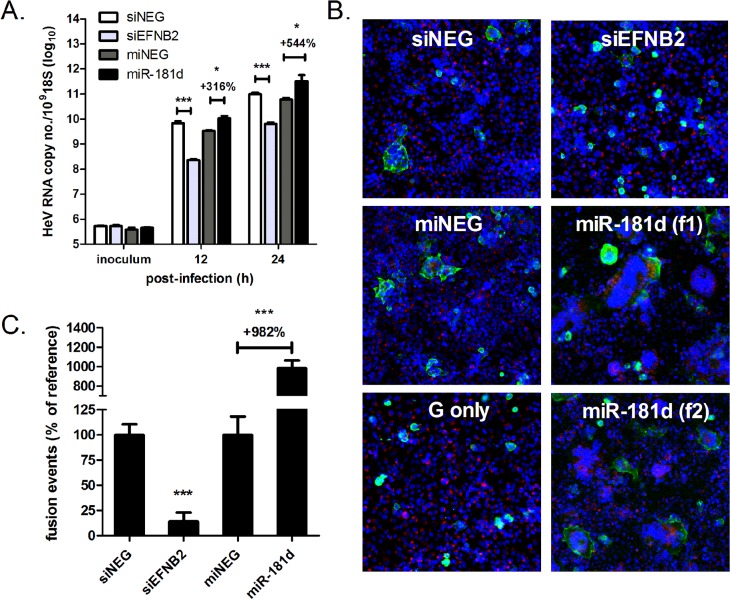
miR-181 significantly enhances HeV RNA synthesis and F- and G-mediated cell-cell fusion. (A) qRT-PCR measurements of intracellular viral RNA copy number in HeLa cells infected with HeV (MOI 5). ***p≤0.001; *p≤0.05. HeV RNA values were normalised to cellular 18S levels. (B) Cell-to-cell fusion of HeV-F and HeV-G-expressing HEK-293T effector cells to HeLa cells treated with indicated siRNA or miRNA agonists. Syncytia were imaged using automated fluorescence microscopy. Nuclei are shown in blue (DAPI), effector cells in green (HeV-G staining) and target cells red (DiO lipid dye). (f1) and (f2) are images of cells transfected with miR-181d from two different microscopy fields. (C) Quantification of fusion events by counting all nuclei present in all syncytia. Values are normalised as a percentage to siNEG or miNEG. *p ≤ 0.05, ***p ≤0.001 compared to respective negative controls.

At 12 h.p.i., a four-log_10_ increase in viral RNA above inocula levels was observed in cells transfected with either miNEG or siNEG (Fig 5A). This increase could be suppressed by siRNA-mediated knockdown of the entry receptor for HeV, ephrin-B2. In contrast, pre-treatment of cells with miR-181d agonists increased the amount of HeV RNA by approximately 3-fold relative to cells treated with control agonist. Thus, miR-181 promotes henipavirus infection at, or prior to, the step of viral RNA synthesis. Similar trends in viral RNA levels amongst the treatment groups were also observed at 24 h.p.i.

### miR-181 significantly enhances HeV F- and G-mediated cell-cell fusion

Changes in viral RNA synthesis as measured by qRT-PCR during a single cycle infection could be due to effects on viral entry, genome replication, viral transcription or translation. To address whether miR-181 promotes entry of henipaviruses, a cell-cell fusion assay was performed using 293T effector cells expressing HeV F and G-glycoproteins [14]. Target cells (HeLa) were pre-transfected with either miR-181d agonist or the control agonist (miNEG), stained with a live cell membrane dye, and co-cultured for 24 h with effector cells expressing both glycoproteins or HeV G alone. Syncytia formation was imaged after fixation of the co-cultures and immunofluorescent staining for surface G glycoproteins. As controls, target cells were pre-treated with either siRNA duplexes targeting ephrin-B2 (positive control for impaired cell entry) or siNEG.

As expected, cells transfected with either siNEG or miNEG formed many multinucleated cells with the effector cells (Fig 5B). In contrast, cells with depleted ephrin-B2 either did not fuse with the effector cells, or fused into smaller syncytia with less nuclei. Additionally, effector cells expressing G singly did not develop syncytia with any of the target cells. Interestingly, miR-181-transfected cells induced substantially more, and larger, syncytia. Cell-cell fusion was so extensive, in a few instances polykaryons with at least 100 nuclei were observed. The extent of fusion for each treatment group was measured using automated image analysis. Results corroborate what was observed by visual inspection, indicating that miR-181 overexpression induced a drastic 9- to 10-fold increase in fusion events relative to control (Fig 5C). Conversely, ephrin-B2 knockdown caused about a 90% reduction in syncytia formation.

### miR-17 promotes henipavirus infection but does not enhance HeV F- and G-mediated cell-cell fusion

Considering the striking pro-fusogenic activity of miR-181, we wondered whether this effect is unique to the miR-181 family of miRNAs. The miR-17~93 family was another high-ranking pro-viral hit from the dual miRNA screens (Figs 1D and 2B). We decided to test the impact of miRNAs from this family on henipavirus-induced cell-cell fusion.

**Fig 6:**
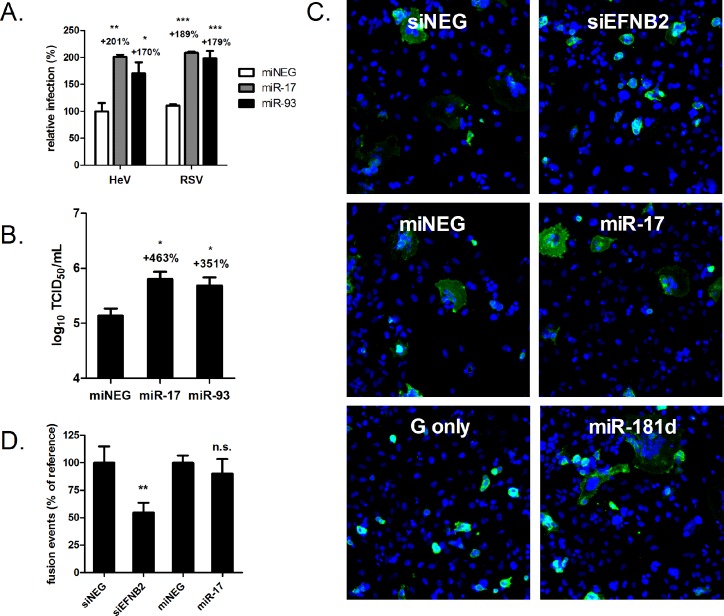
miR-17 promotes henipavirus infection but does not enhance HeV F- and G-mediated cell-cell fusion. (A) Percentage of cells infected with HeV or RSV (24 h, MOI 1), after 72 h transfection with miR-17, miR-93 or control agonists. ***p≤0.001, **p≤0.01, *p≤0.05 compared to control agonist. (B) TCID_50_ virus titres of supernatants derived from HeLa cells infected with HeV for 24 h (MOI 1), at 72 h post-transfection with agonists. *p≤0.05 compared to control agonist. (C) Cell-cell fusion of HeV-F and -G expressing HEK-293T cells to HeLa cells treated with indicated siRNA or miRNA agonists. Syncytia were imaged using automated fluorescence microscopy. Nuclei are shown in blue (DAPI) and effector cells in green (HeV-G staining). (D) Quantification of fusion events by counting of all nuclei present in all syncytia. Values are normalised as a percentage to siNEG or control agonist. n.s. not significant; *p≤0.05, **p≤0.01; ***p≤0.001 compared to respective negative controls.

We first sought to validate the pro-viral effects of the miR-17~93 family using wild-type HeV. Agonists for two representative members of the miR-17~93 family, miR-17 and miR-93, were transfected into HeLa cells, and the permissiveness of these cells to HeV infection was evaluated using quantitative immunofluorescent analysis. The proportion of infected cells was 2-fold higher in the cells treated with miR-17 agonists compared to cells treated with control agonists (Fig 6A). This was commensurate with the uptick in infection ratio in the miR-93-treated cells (170% relative to control). Virus yields in the supernatants of miR-17 agonist-treated cells were also enhanced, as measured by TCID_50_ assays (Fig 6B). For comparison, the activity of these agonists on another paramyxovirus, RSV, was also analysed using quantitative immunofluorescence microscopy (Fig 6A). Akin to the impact of miR-17~93 on HeV infection, we found that the ratios of infected cells were also significantly higher in the miR-17 and miR-93 agonist transfected cells (189% and 180% of control, respectively). These results further validate the datasets from the complementary screens (Fig 1), and indicate that members of the miR-17~93 family are indeed promotive for wild-type henipavirus infection. However, and rather intriguingly, unlike miR-181 (Fig 4), members of the miR-17~93 family appear to also exhibit pro-viral effects on a paramyxovirus from a different subfamily than the henipaviruses.

Once the pro-viral ability of miR-17 was established, we then performed the cell-cell fusion assay to assess the fusogenic effects of miR-17 agonists. Consistent with our previous observations (Fig 5B), target cells depleted of ephrin-B2 exhibited muted fusion activity relative to control cells (Fig 6C). In contrast, target cells treated with miR-181d agonists formed larger multinucleated cells. Interestingly, even though miR-17 enhanced HeV infection, cells loaded with miR-17 agonists did not fuse at significantly higher efficiencies than negative control cells. Of note, smaller syncytia were formed in this experiment as less target cells were added to the co-cultures. The extent of syncytia formation in the captured images of all microscopy fields were additionally quantified using automated image analysis software (Fig 6D).

In sum, these results indicate that HeV-glycoprotein mediated cell-cell fusion is greatly stimulated by miR-181, but not by miR-17, suggesting that miR-181 specifically facilitates henipavirus infection by enhancing host entry and, quite possibly, by supporting cell-to-cell spread during late stages of infection via syncytia formation.

### Expression of entry receptors ephrin-B2/B3 and viral fusion glycoproteins are not appreciably enhanced by miR-181

Since miR-181 specifically promotes infection of henipaviruses but not other paramyxoviruses, it is quite likely miR-181 increases membrane fusion by directly targeting viral and/or host molecules unique to the henipavirus fusion machinery. Although most miRNAs reduce expression of its target mRNA, there have been instances where miRNA binding improves stability and translation of the mRNA [30, 31]. Thus, we next investigated if miR-181 overexpression would enhance expression of the virus entry receptors ephrin-B2 and -B3, as well as the viral fusion glycoproteins F and G. miR-181a agonists were included in this analysis, subsequent to the validation of their pro-fusion nature in infection and fusion assays (S2 Fig). Perhaps surprisingly, S3 Fig shows that expression levels of the host and viral molecules which are known to be directly involved in fusion were not appreciably boosted by miR-181d. Similar results were observed for miR-181a as well. In fact, miR-181 downregulated ephrin-B3 and and HeV F glycoprotein by about 30 to 40%.

### A subset of class A Eph receptors contain putative miR-181 target sites

Given the substantial impact of miR-181 on cell-cell fusion (Fig 5B and 5C), it was intriguing that the miRNA did not considerably enhance expression levels of host and viral molecules known to be involved in entry and fusion. This led us to hypothesize that miR-181 supported fusion by down-regulating the expression of one or more novel cellular factor(s) that antagonizes expression and/or activity of the henipavirus entry receptors, ephrin-B2 or–B3. In the host, ephrins serve as native ligands for cellular Eph receptors (Ephs), which are high-affinity cell surface receptors belonging to the receptor tyrosine kinase family. Ephs and ephrins are further subdivided into classes A and B. Co-crystal structures of ephrin-B2 in complex either with its natural Eph [32, 33] or with the henipavirus G glycoproteins [34] have been solved. Intriguingly, these structures reveal the GH binding loop of ephrin-B2 to be the same dynamic region predominantly responsible for mediating the binding of ephrin-B2 to its natural Eph receptors as well as to the viral attachment proteins (S4A Fig.) [35]. Because the affinities of ephrin-B2-HeV-G and known ephrin-B2-Eph receptor interactions are within comparable nanomolar range [34, 36, 37], it implies that the Ephs may compete with the viral glycoprotein for binding to ephrin-B2. If true, one would expect Ephs to exhibit anti-viral properties, and any miRNAs which target these receptors for knockdown would be pro-viral.

Indeed, Bossart et al. previously reported that soluble forms of Ephs (EphB3 and B4) can compete with henipavirus G proteins for binding to ephrin-B2 or ephrin-B3, and can also inhibit virus infection [38]. Additionally, analysis of data from our recently published genome-wide siRNA screen [14] reveal that Ephs are more likely to be inhibitors of HeV infection (S4B Fig and S4 Table). That said, algorithmic analysis by TargetScan [39, 40] of all human Ephs does not predict miR-181 binding sites in the 3’ UTR of the mRNAs of EphB3 or EphB4 (S4 Table). Of the three Ephs which have putative binding sites, all belong to class A receptors. This includes the EphA5 receptor, which is the most anti-viral Eph receptor in our published RNAi screen (S4B Fig). Even though ephrin-B2 or ephrin-B3 tend to preferentially engage with class B Eph receptors (e.g. EphB3 and B4), there is some precedence for crosstalk interaction with class A Eph receptors as well, such as EphA4 [41] and EphA3 [42] (S4A Fig). Thus, a potential model for the pro-viral mechanism of miR-181 posits that the miRNA down-regulates expression of Ephs, increasing the pool of unbound ephrin-B2 or ephrin-B3 for henipavirus G glycoproteins to attach and trigger entry/membrane fusion.

### EphA5 and EphA7 are novel negative regulators of henipavirus fusion

To test this model, we began by assessing the impact of silencing select Ephs on HeV infection. We opted to assess all Ephs with putative miR-181 target sites as predicted by TargetScan [39, 40], namely EphA4, A5 and A7 (S4 Table). Even though it was not predicted to contain any miR-181 binding sites, EphB4 was the most anti-viral hit of the class B receptors in the RNAi screen and was previously shown to compete with HeV G glycoprotein for ephrin-B2 binding [38], so it was incorporated into our study as well.

**Fig 7:**
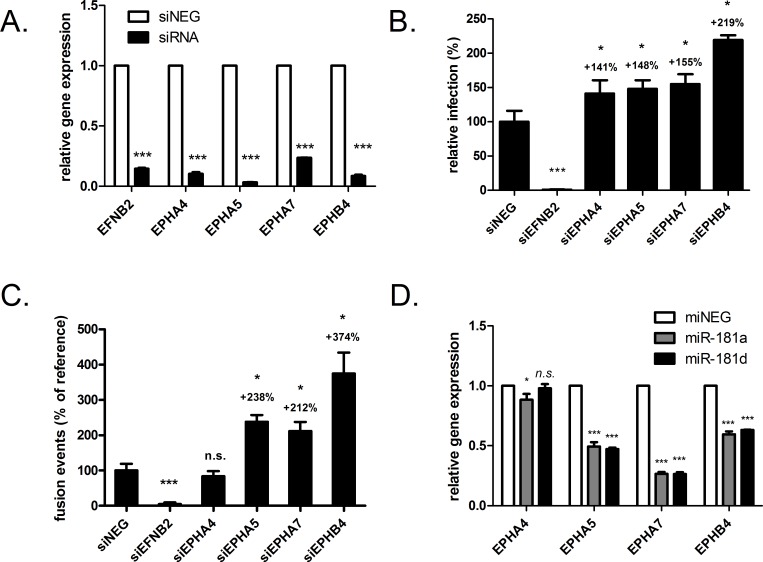
Select Eph receptors inhibit HeV infection and cell-cell-fusion and are miR-181 target genes. (A) Relative mRNA levels of indicated target genes in HeLa cells 72 h post-transfection with siRNAs (40 nM). ***p<0.001 compared to siNEG (B) Relative percentage of cells infected with HeV (24 h, MOI 0.5), after 72 h transfection with siRNAs targeting indicated molecules. *p<0.05, ***p<0.001 compared to siNEG. (C) Relative mRNA levels of Eph receptors A4, A5, A7 and B4 in HeLa cells, 72 h post transfection with miRNA agonists (25 nM). N.s. not significant; **p<0.01, ***p≤0.001, compared to control agonist. (D) Cell-cell fusion of HeV-F and–G expressing HEK-293T cells to HeLa cells treated with indicated siRNAs. Values are normalised as a percentage to siNEG or control agonist.

We first validated the siRNAs used to silence expression of the particular Ephs. Transfecting HeLa cells with siRNAs targeting EphA4, A5, A7 or B4 resulted in a >80% relative decrease in target mRNA expression (Fig 7A). Down-regulating EphA4, A5, A7 or B4 with these siRNA duplexes all caused significant increases in HeV infection (Fig 7B), with EphB4 exhibiting the greatest impact (~200% of siNEG). We next performed a cell-cell fusion assay to directly test the effects of silencing of these receptors on henipavirus fusion. Interestingly, even though EphA4 was inhibitory for virus infection, it did not seem to be repress syncytia formation (Fig 7C), suggesting that EphA4 blocks infection at a step post entry. On the other hand, EphA5, A7 and B4 all suppressed cell fusion, with relative trends comparable to that seen in the infection assay (Fig 7B). We therefore demonstrate, for the first time, that in addition to class B receptors (i.e. EphB3 and B4), class A Eph receptors can also inhibit henipavirus cell-cell fusion.

### miR-181 targets EphA5, EphA7 and EphB4, but not EphA4, for downregulation

To evaluate whether these anti-fusion Ephs are suppression targets of miR-181, the mRNA levels of the Ephs in agonist-transfected cells were measured by qRT-PCR. Over-expressing miR-181a and miR-181d caused a significant reduction in EphA5 and A7 mRNA levels, with the latter showing the greatest reduction in gene expression (Fig 7D). In contrast, EphA4 levels were not impacted by miR-181d, and were only modestly (11%) affected by miR-181a, demonstrating some level of specificity in the regulation of Eph receptor expression by miR-181. Interestingly, although it was not computationally predicted to have any target site in its mRNA 3’ UTR, EphB4 levels were reduced by both miRNAs by about 40% (Fig 7D). These data show that endogenous levels of the henipavirus fusion regulators EphA5, A7 and B4 can all be significantly suppressed by miR-181 expression. Simultaneous suppression of all three negative fusion regulators would conceivably result in a cellular state with abundant unbound ephrin molecules, strongly favoring efficient activation of the viral fusion machinery. This provides a coherent mechanistic model for how miR-181 may expedite host entry and virus spread during infection.

### Expression of miR-181 is up-regulated in circulating biofluids derived from *in vivo* models of henipavirus disease

Previous studies have reported that members of the miR-181 family are involved in different aspects of immune regulation [43–45]. In particular, miR-181a expression levels have been shown to correlate with pro-inflammatory signals (e.g. IL-1β, IL-6, and TNF-α) in blood tissues of humans with chronic inflammation, as well as blood of LPS-treated mice [46]. Additionally, miR-181 expression in human kidney tissues were found to be associated with increased transcription of genes of inflammation pathways [47]. As the biological relevance of miRNAs is linked to their prevalence [48], we considered examining changes in levels of miR-181 molecules in *in vivo* infection models for henipaviruses. We hypothesized that, in conjunction with the host pro-inflammatory response during early infection, miR-181 expression might be up-regulated in HeV-infected mammals, but perhaps the virus co-opts this up-regulation to support infection and viral spread in the host.

**Fig 8:**
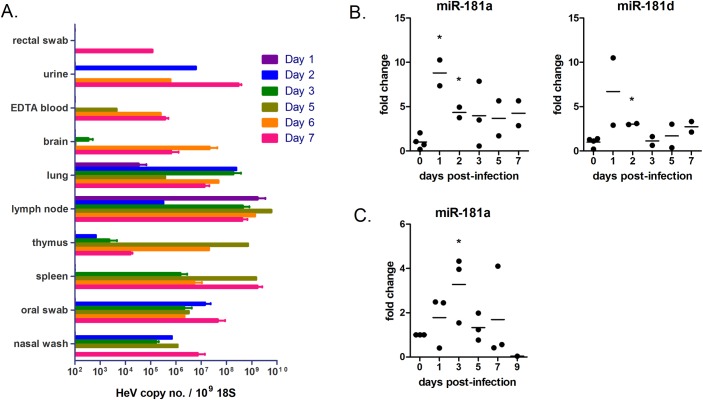
Expression levels of miR-181 in biofluids of animals infected with HeV are increased. Sixteen ferrets were infected with HeV at BSL-4. At predetermined time-points, ten different tissues were harvested and analysed for viral RNA loads by qRT-PCR (A). (B) qRT-PCR analysis of miR-181d levels in the serum samples of the ferrets. Values were normalized to the U6 RNA. *p≤0.05 compared to day 0. (C) qRT-PCR analysis of miR-181d in blood of horses from a published HeV infection study [52].

Ferrets have been established as an animal model for the study of several human respiratory viruses [49], including Hendra and Nipah viruses [50, 51]. Accordingly, sixteen adult ferrets were exposed to HeV via the oronasal route, monitored for clinical signs and play activity, and two or four ferrets were euthanized at 1, 2, 3, 5, 6 and 7 day post-exposure (d.p.e.). After an incubation period of about five days, some ferrets started exhibiting weight loss, which correlated with an increase in rectal temperatures (S5A Fig and S5B Fig). By seven d.p.e., visible clinical signs, including depression, lack of grooming, or a decrease in playfulness, were observed in three out of four remaining ferrets. Establishment of HeV infection was confirmed by performing qRT-PCR for HeV genomic RNA on ten different tissues from the ferrets (Fig 8A) as well as by virus isolations (S5C Fig). By one d.p.e., viral RNA was detected in the retropharyngeal lymph nodes and in lung tissues, and by three d.p.e., HeV RNA was also recovered from the spleen, thymus and brain, suggesting neurotrophic spread characteristic of henipavirus disease. The higher viral RNA loads observed in the lung, lymph nodes and spleen is congruent with previous HeV infection studies performed in ferrets [50]. We next purified total small RNA from serum samples (from all days except for day six), and then performed qRT-PCR using a primer specific for miR-181a and miR-181d. We found that, as early as one d.p.e., miR-181a and miR-181d became elevated in the serum of these ferrets during the course of infection (Fig 8B). Reminiscent of what was observed in mice treated with LPS [46], this early up-regulation appears to be transient, as by day three, levels of miR-181a and miR-181d began dropping to baseline levels.

Natural HeV outbreaks occur in horses, causing severe febrile illness associated with respiratory and neurologic signs. Accordingly, horses serve as another animal model for respiratory and neurologic HeV disease. A HeV infection study involving three experimental horses was previously described [52]. Here, total small RNA was purified from the stored blood samples of these horses, and the relative expression of miR-181a and miR-181d on 0, 1, 3, 5, 7 and 9 d.p.e. were determined by qRT-PCR (Fig 8C). Similar to results observed in ferret, transient yet significant increases in circulating miR-181 molecules were observed during the early stages of infection. Collectively, these observations demonstrate in two different *in vivo* models that members of the miR-181 family are up-regulated early in the host during HeV infection, implicating a biological role for miR-181 in host immunity as well as in henipavirus pathogenesis.

## Discussion

The development of novel therapeutics for viruses of clinical significance relies on our knowledge of the dynamic interplay between the virus and the human host, and our ability to apply such knowledge to disrupt the viral dependence on host factors. However, progress in our understanding of virus-host interactions of many deadly viruses of significant public health importance (e.g. Ebola, MERS, Nipah virus) is hampered by the high-cost and technical challenges associated with studying these viruses under BSL-3 or BSL-4 conditions. To circumvent these issues, different strategies and approaches have been developed, such as the use of pseudotyped particles [53] or minigenome assays [54]. These approaches, though of much utility, have their shortcomings; in particular, they cannot fully reproduce the entire life cycle of the virus. Therefore, significant progress still needs to be made towards the development and validation of our capabilities to perform technically-challenging experiments in high biocontainment environments.

In recent years functional genomics has become a popular research approach for unbiased discoveries of novel genes and molecular pathways involved in a particular biological process. For infectious diseases, functional genomics has demonstrated much power in its ability to dissect the dynamic interplay between host and viral factors during a virus infection, paving the way for novel drug targets. For instance, a haploid genetic screen resulted in the discovery of the once elusive entry receptor for Ebola virus [55]. That said, methods used in functional genomics, such as high-throughput RNAi screens, are technically challenging and laborious, especially at BSL-4.

In this report, we screened 834 host miRNAs, using both engineered agonists and antagonists, for their ability to enhance or inhibit infection of HeV in human cells. As two complementary screens were performed, we exploited this duality and cross-referenced the two screens to increase our confidence in the top hits. Both complementary screens converged on members of four miRNA families (miR-181, miR-17~93, miR-520h, miR-548d) that strongly promoted henipavirus infection. Since all four members of the miR-181 family were pro-viral hits using this approach, we focused our validation efforts on miR-181. We show that miR-181 promoted infection of both wild-type HeV and NiV infections. Interestingly, this infection enhancement seems to be primarily mediated via the ability of miR-181 to significantly augment henipavirus glycoprotein-mediated cell-cell fusion, implicating miR-181 in the enhancement of henipavirus entry. Congruent with this notion, viral RNA synthesis in a single round of infection is elevated in cells transfected with miR-181 agonists. This pro-fusion effect is specific to the miR-181 family, as transfection with agonists of another strongly pro-viral miRNA (miR-17), did not appreciably alter syncytia formation. Since henipavirus mediated cell-cell fusion is both a surrogate model for virus entry as well as a natural phenomenon during late stages of infection, it is likely that in addition to enhancing henipavirus entry, miR-181 also promotes more efficient cell-to-cell spread of this virus by merging the cytosols of neighbouring cells more rapidly. To our knowledge, this is the first instance of subversion of a host miRNA by a virus to promote entry and membrane fusion.

Investigation into the pro-fusion mechanism of miR-181 led us to hypothesize that Eph receptors, the cellular binding partners of the henipavirus entry mediators ephrin-B2 and -B3, may act as potent anti-fusion regulators. Eph receptors and their ephrin ligands are involved in intracellular signalling via direct cell-cell contact and receptor-ligand interactions [56]. These molecules are divided into two different classes (A and B), and Ephs of a particular class tend to bind to ephrins of the corresponding class [57]. Exceptions to the rule exist, such as the ability of EphB2 to bind to ephrin-A5 [58]. Initial *in silico* analysis revealed that EphA4, EphA5 and EphA7 possess putative miR-181 binding sites in the 3’ UTRs of their transcripts. Our experiments subsequently showed that EphA5 and EphA7, but not EphA4, are novel suppressors of the fusiogenic effects of henipavirus glycoproteins. Importantly, levels of EphA5 and EphA7, but not EphA4, are reduced by overexpression of both miR-181a and miR-181d, indicating that these class A Ephs are target genes for the miR-181 family, and that the pro-fusion phenotype of miR-181 are, at least in part, due to its downregulation of specific class A Ephs.

We also demonstrated, using an approach different from a previous study [38], that EphB4, an Eph receptor from the same class as the henipavirus entry receptors, has potent anti-fusion characteristics. Indeed, at comparable knockdown levels, siRNAs to EphB4 increased syncytia formation significantly more than siRNAs to EphA5 or EphA7 (Fig 7A and 7C). This data also lends support to the notion originally proposed by Bossart et al. (2008) that class B Ephs can compete with henipavirus glycoproteins for binding to entry receptors to hamper virus entry. Intriguingly, even though the 3’UTR of EphB4 transcripts does not contain any sequence that is complementary to the seed region of miR-181, EphB4 levels were downregulated by miR-181 expression. Though less commonly reported, miRNAs can modulate gene expression by binding to the coding region of mRNAs [59]. Accordingly, human EphB4 does contain a putative miR-181 binding site in its ORF, providing an avenue for miR-181 regulation of its expression.

Since EphA5 and EphA7 do not interact with class B ephrins [56], it is likely the effects of downregulated EphA5 and EphA7 on promoting henipavirus fusion is indirect. One possibility is that since some crosstalk already exists between the two classes of the Eph-ephrin interaction network (e.g. EphB2 with ephrin-A5 [58]) [57], downregulation of some molecules in the network may have broader, indirect effects on the availability of other molecules in the system, including the ephrins utilized by henipaviruses. For instance, repression of EphA5 and EphA7 expression will free up more ephrin-A molecules, including ephrin-A5. The increased level of unbound ephrin-A5 results in its sequestration of EphB2 and thereby make more ephrin-B2 and ephrin-B3 molecules available for binding by the henipavirus glycoproteins.

Alternatively, EphA5 and EphA7 may act directly on ephrin-B2 and B3 to regulate fusion, but via lateral *cis* interactions on the same cell. It was more recently shown that the canonical intra-class binding rule may only apply for *trans* interactions [42]. For example, via lateral *cis* interactions, ephrin-B2 can attenuate the *trans* ligand-binding capacity of EphA3 and its activation, even though ephrin-B2 does not bind to EphA3 by canonical *trans* interaction [42]. This *cis* inhibition of EphA3 by ephrin-B2 implies that *cis* interactions do not exhibit the same receptor-ligand selectivity as *trans* interactions, providing a possible non-canonical mechanism for EphA5 and EphA7 to modulate ephrin-B2 activity.

Collectively, our data supports a model where simultaneous inhibition of multiple anti-fusion Ephs from both receptor classes by miR-181 contributes to greatly enhanced membrane fusion and infection. Considering that EphB4 is most antagonistic towards fusion (Fig 7C), it likely makes the most significant contribution to the pro-fusion phenotype of miR-181. Though this is the first report of a host miRNA that promotes virus fusion by suppressing anti-fusion receptors, there is some precedence for host receptor-ligand interactions that negatively regulate virus entry. Natural host ligands (i.e. BTLA and LTα) of the herpes simplex virus entry receptor, HVEM, can inhibit binding of the virus glycoprotein gD to HVEM and suppress infection [60]. Additionally, nectin members can compete with the measles virus hemagglutinin glycoprotein for binding to its exit receptor nectin-4 [61, 62].

Even though the role of miR-181 in inflammation and NKT-cell maturation has been documented [23, 43–46], little has been reported about its role in the infection of other viruses. In stark contrast to henipaviruses, miR-181 has been shown to be inhibit infection of porcine reproductive and respiratory syndrome virus [63]. Interestingly, this inhibition occurs at PRRSV host entry and is achieved by targeting the entry receptor CD163 for downregulation [64].

We found that miR-181 did not affect infection by paramyxoviruses from other genera, indicating specificity in the henipavirus-miR181 virus-host interaction. This supports the model that miR-181 enhances syncytia formation by targeting Ephs that naturally associate with the henipavirus entry receptors ephrin-B2 and B3. On the other hand, miR-17 enhances the infection of HeV as well as RSV, suggesting that the pro-viral effects of miR-17 are broadly applied to the paramyxovirus family, and perhaps beyond this family. For instance, miR-17 has recently been shown to be critical for the replication of pestiviruses, primarily via enhancing viral translation and vRNA stability [12]. Cross-referencing of results from the siRNA screen of host genes associated with HeV infection suggests that miR-181 and miR-17~93 target multiple host genes which are anti-viral for HeV, and that the net outcome of cellular expression of miR-181 or miR-17~93 is likely a host microenvironment that is more conducive for henipavirus infection. These results indicate that, in addition to its role in regulating fusion, miR-181 might act via other anti-viral host mediators to induce a situation that is broadly supportive of henipavirus replication. Consistent with this, we observed that miR-181 is up-regulated in sera of ferrets and blood of horses as early as day 1 during a henipavirus infection. It is tempting to speculate that the host pro-inflammatory response (of which blood miR-181 is correlated with) promotes the early phase of virus spread in the host, thereby contributing to disease progression and pathogenesis [43, 44, 46]. Along similar lines, but in a chronic infection, serum miR-181b is positively correlated with hepatitis B virus (HBV) DNA levels in human patients, and with disease progression of chronic HBV infection [65]. The model of miR-181-mediated immune pathogenesis has potential implications for risk factors associated with susceptibility to henipavirus disease, as well as for the strategic design and development of novel immunotherapy for henipavirus infections.

Direct binding interactions between host miRNAs and viral genomes have been reported for certain RNA viruses like EEEV and pestivirus [11, 12]. These interactions often have functional relevance in terms of supporting viral replication. It was recently first demonstrated that the functional RNAi protein Argonaute preferentially associates with certain subgenomic segments of paramyxoviruses as well [12]. In particular, a higher abundance of Argonaute association with M and N segments of the hMPV RNA was observed relative to the L segment. Due to limited resolution in the AGO-CLIP analysis in that study, whether these physical interactions involve specific host miRNAs remains to be addressed. Considering that our complementary screens identified multiple miRNAs that support henipavirus infection, an interesting subject for future studies would be to investigate whether the pro-viral miRNAs which we identified here bind to the genomes of paramyxoviruses, and whether such interactions have functional roles in supporting infection.

In summary, these dual screens further the understanding of the role of host-derived small noncoding RNAs in the infection cycle of henipaviruses, and provide a miRNA-based resource for the study of viruses from the order mononegavirales, including members of both the filovirus and paramyxovirus families, which presents significant threats to human and animal health. This study implicates miR-181 and certain class A Eph receptors as critical modulators of henipavirus membrane fusion, and highlights how the natural innate immune response of the host can be exploited by a RNA virus to promote cell-to-cell spread.

## Materials and Methods

### Cell culture

HeLa cells (*ATCC* CCL-2) and African green monkey kidney epithelial Vero cells (*ATCC* CRL-81) were maintained in growth medium comprised of DMEM GlutaMAX supplemented with 10% (v/v) fetal calf serum and 100 U/mL penicillin/streptomycin. Madin-Darby Canine Kidney (MDCK) cells (*ATCC* CCL-34) and HEK 293T cells (*ATCC* CRL-3216) were maintained in growth medium comprised of RPMI 1640 medium supplemented with 10% (v/v) fetal calf serum, 10 mM HEPES, 2 mM L-glutamine, 1 mM MEM sodium pyruvate and 100 U/mL penicillin/streptomycin. All cells were incubated at 37°C in a humidified atmosphere containing 5% CO_2_.

### Viruses

All virology work was conducted at the CSIRO Australian Animal Health Laboratory. Recombinant HeV, wild-type HeV (both Hendra virus/horse/1994/Hendra), NiV (Nipah virus/Malaysia/human/99), MeV (wild type Edmonston strain), MuV (Enders strain) and RSV (strain A2) were passaged in Vero cells. Influenza A/WSN/33 (H1N1) (kind gift, Professor Lorena Brown, University of Melbourne) was passaged in the allantoic fluid of 10-day embryonated specific pathogen-free chicken eggs. HeV and NiV were handled under BSL-4 conditions, MeV and MuV at BSL-3, and RSV and influenza A/WSN/33 at BSL-2. All viruses were aliquoted and stored at −80°C for inoculations.

### High-throughput microRNA screening

High throughput miRNA agonist and antagonist screens were performed largely as described [14]. Briefly, HeLa cells were transfected in 384 well plates with miRIDIAN miRNA agonist and antagonist (final concentration 25 nM) libraries using DharmaFECT (DF) 1 lipid transfection reagent (Dharmacon RNAi Technologies, GE, Lafayette, Colorado, USA). Genome-wide miRNA libraries (catalog numbers in S1 Table and S2 Table) were screened at the Victorian Centre for Functional Genomics (VCFG). At 72 h post transfection, in parallel with the point of HeV infection, cell viability for each well was assessed by fixing (4% paraformaldehyde for 10 min) and staining plates with the nuclear stain 4',6-Diamidino-2-Phenylindole, Dihydrochloride (DAPI) (Invitrogen, Carlsbad, CA; 1 μg/ml for 20 min in phosphate buffered saline). HeV infection was quantitated in separate plates suitable for luminescence assays. 72 h post-transfection, cells were infected with recombinant HeV (MOI 0.1 using a BioTek 406 liquid handler housed in a class II biosafety cabinet at BSL-4. At 24 hours post-infection, media was removed and 20 μL of PBS added per well. Luminescence was then measured by addition of 20 μL of Bright-Glo Luciferase reagent (Promega, Madison, WI) and reading on a Synergy H4 multimode microplate reader (BioTek).

### Bioinformatic analysis of screen data

Data analysis was performed as described [14]. The experimental robustness was evaluated for each screened plate using the Z’ factor calculation [20], comparing the negative control (siNEG), positive control (siLUC) and death control (siPLK1) for both cell viability and HeV infection. Robust z score = (sample value-sample median)/sample median absolute deviation was used as the hit identification method [20, 66].

### Transfections

For work subsequent to the miRNA screens, HeLa cells were seeded overnight in 96-well plates (8 × 10^3^ cells/well) in growth medium. The following day, growth medium was replaced with antibiotic-free medium (DMEM with GlutaMAX, 10% (v/v) foetal calf serum) (100 μL/well) before cells were transfected with miRNA agonists at a final concentration of 25 nM. Cells were then incubated at 37°C for 72 h (media was changed to growth media at 24 h-post transfection).

### Tissue culture infective dose (TCID_50_) analysis

TCID_50_ assays were performed as described [22]. Infectious virus titre was then calculated in accordance with the method described by Reed and Muench [67].

### Influenza virus plaque assay

MDCK cells were seeded in 6-well plates (1 x 10^6^ cells/well) in growth media. The following day, cells were washed with PBS and media was changed in order to ensure the removal of any detached cells. When cell confluency reached 100%, 10-fold serial dilutions of influenza virus stocks in FCS-free media (RPMI 1640, 10 mM HEPES, 2 mM L-glutamine, 1 mM MEM sodium pyruvate, 100 U/mL penicillin/streptomycin) were prepared. Cells were then washed with FCS-free media, infected with 500 μL of the appropriate viral dilution and incubated at 37°C for 45 minutes, with gentle shaking every 15 minutes. 2 × L15 medium was then mixed with 1.8% (w/v) pre-autoclaved agarose and added to cells. Cells were incubated at 37°C for 3 days. Following incubation, cells were fixed with 5% (v/v) formaldehyde for 1 h. The agarose overlay was then removed and cells were stained with 0.1% (w/v) crystal violet, diluted in 4% (v/v) ethanol. After 10 minutes of staining, cell monolayers were rinsed with water and visible plaques were counted by eye.

### Immunofluorescence microscopy

Immunofluorescence microscopy was performed largely as described [14]. Cells were fixed with 4% (w/v) paraformaldehyde, stained with primary antibodies to viral antigens and analysed using an automated Thermo Fisher Scientific CellInsight Imaging System. HeLa cells in 96 well plates were imaged at a magnification of 10 x, 49 fields/well representing the entire well. The percentage of infected cells was quantified using the Target Activation bioapplication of the Cellomics Scan software and was determined by dividing the number of antigen-positive cells by the total cell number, multiplied by 100.

### Cell-cell fusion assay

HeV-F and -G mediated fusion assays were performed as described previously [14].

### Hendra virus qRT-PCR

Quantitative RT-PCR for HeV RNA was performed as described previously [14].

### Quantitative real-time PCR for Eph-ephrin and HeV

For the Eph-ephrin experiments, total RNA was purified from agonist or siRNA-transfected HeLa cells using the RNeasy Plus RNA purification kit from Qiagen (USA) and stored at −80°C. cDNA synthesis was performed using Superscript III reverse transcriptase kit (Invitrogen) according to the manufacturer's guidelines. qRT-PCR was performed using SYBR green (Invitrogen) on a StepOnePlus Real-Time PCR System (Applied Biosystems). PCR cycling for gene detection was at 95°C for 10 min, followed by 40 cycles of 95°C for 15 s and 60°C for 1 min. A melting curve analysis was performed to check for primer-dimer artifacts and to verify assay specificity. PCR primers were purchased from GeneWorks Ltd (Adelaide, Australia). Data were analyzed using the ΔΔCT method and were normalized to GAPDH for mRNA detection. qRT-PCR for HeV RNA during the infection time-course experiments were performed as described previously [14].

### Ethics statement

All animal studies were approved by the CSIRO Australian Animal Health Laboratory’s Animal Ethics Committee (document #1568) and conducted following the Australian National Health and Medical Research Council Code of Practice for the Care and Use of Animals for Scientific Purposes guidelines for housing and care of laboratory animals.

### Ferret infection study

Sixteen ferrets (aged 12–18 months) were exposed to 5000 TCID_50_ of Hendra virus/Australia/Horse/2008/Redlands by the oronasal route as previously described [50]. Prior to any manipulations, animals were immobilised with a mixture of ketamine HCl (3 mg/kg) and medetomidine (30 μg/kg); atimepazole was administered for reversal at 50% of the medetomidine dose. After virus exposure, animals were monitored for changes in play activity, other clinical signs of disease, and fever. They were randomly assigned to euthanasia on post-exposure days 1, 2, 3, 5, 6 or 7, when clinical samples including nasal washes, mucosal swabs, blood and urine were collected together with multiple tissue specimens. qRT-PCR and viral loads from the tissues were assessed as described previously [50].

### Isolation of RNA from ferret and horse biofluids

EDTA blood samples from horse infected with HeV were derived from an experimental time-series trial [52]. Ferret sera were collected as described above. Total RNA (including small RNAs) was harvested using Tri-reagent (Sigma, St. Louis, MO) following manufacturer's instructions. Phase separation was achieved by adding 200 μL chloroform to each tube, shaking vigorously and incubating samples for a further 3 min at room temperature, prior to centrifugation at 12,000 x *g* for 15 min at 4°C. The aqueous upper phase of each sample was then placed in a new tube, before the addition of 10 μg (0.5 μL) of glycogen and 250 μL of isopropanol. Samples were incubated for 10 min at room temperature prior to being centrifuged at 12,000 x *g* for 10 min at 4°C. The supernatants were then removed, and each RNA pellet was washed with 500 μL 75% (v/v) ethanol before being centrifuged at 7500 x *g* for 5 min at 4°C. RNA was then resuspended in 20 μL of RNase-free water.

### qRT-PCR analysis of miR-181 expression

Prior to cDNA synthesis, RNA samples (1 μg) were treated with RNase-free DNase according to manufacturer’s instructions (Promega). DNase-treated RNA was reverse transcribed into cDNA using the Exiqon miRCURY LNA Universal RT miRNA PCR kit, according to manufacturer’s instructions. qRT-PCR preparation was also performed using the Exiqon miRCURY LNA Universal RT miRNA PCR kit, which included miR-181 and U6primers. For each reaction, 5 μL PCR Master Mix and 1 μL PCR Primer Mix were added to 4 μL (5 ng) of the diluted cDNA template. qRT-PCR analysis was performed on the Applied Biosystems 7500 FAST Real-Time PCR System. Cycling conditions began with 10 min at 95°C, followed by 40 cycles of 95°C for 10 seconds and 60°C for 1 minute. Following qRT-PCR, miR-181 expression was analysed using the ΔΔC_T_ method and normalised to U6.

### Statistics

All statistical analyses were performed using GraphPad Prism 5 software. The difference between treatment and control groups was analysed using a two-tailed Student’s *t* test, with a *P* value of <0.05 considered to be statistically significant. Error bars represent standard deviations, and all data points are the average of a minimum of 3 replicates.

## Supporting Information

S1 Table Impact of microRNA agonists on HeV infection. (XLSX)

S2 Table Impact of microRNA antagonists on HeV infection. (XLSX)

S3 Table Experimentally validated target genes for miR-181 and miR-17 families (miRTarBase, and their corresponding impact on HeV infection). (XLSX)

S4 Table Impact of members of Eph receptor family on HeV infection [14] and their putative miR-181 binding sites (TargetScan). (XLSX)

S1 Fig Completion of a single cycle of HeV infection at between 12 and 24 hpi. siRNA targeting human EFNB2 or siNEG were transfected into HeLa cells using Dharmafect. 72 hrs after transfection, cells were infected with HeV (MOI 5). At 0, 12 and 24 h.p.i., cell supernatant were harvested and TCID_50_ analysis were performed. a: p≤0.01 compared to inoculum, ***: p≤0.001 compared to 24 h.p.i. siNEG.
(TIF)

S2 Fig miR-181a promotes HeV infection and cell-cell fusion. (A) Percentage of HeLa cells stained positive for HeV-P during HeV infection (24 h, MOI 1), 72 h post-transfection with miNEG or miR-181a agonist (25 nM). (B) Relative fusion events in HeLa cells treated with indicated miRNA agonists.
(TIF)

S3 Fig **Expression levels of HeV entry receptors ephrin-B2 and–B3 and HeV fusion glycoproteins are minimally affected by mR-181** (A) Relative mRNA levels of EFNB2 and EFNB3 in HeLa cells treated with indicated microRNA agonists (25 nM) for 72 h. n.s. not significant; *p<0.05 compared to miNEG (B) HeV-F and -G protein expression in HeLa cells transfected with cDNA encoding HeV-F and HeV-G (100 ng), in the presence or absence of indicated microRNA agonists (24 h transfection, 25 nM). Quantification of HeV-F and–G is shown numerically relative to GAPDH protein expression levels.
(TIF)

S4 Fig Cellular Eph receptors antagonize HeV entry by competing with viral attachment glycoprotein for binding to ephrin-B2. (A) Solved co-crystal structures of ephrin-B2 (blue) in complex with its cellular (EphB2, EphA4) and viral (HeV-G) binding partners indicate that the partners interact with ephrin-B2 primarily via the same binding site on ephrin-B2, the GH loop (red). EphB2 [68] is shown in grey, EphA4 [41] in orange, and viral G-glycoprotein [34] in green. (B) Robust Z scores for all Eph receptors tested in our recently published genome-wide siRNA screen [14].
(TIF)

S5 Fig Changes in health metrics and viral loads of ferrets infected with Hendra virus. The weight (A) and rectal temperatures (B) of the ferrets were recorded daily through the HeV infection trial. (C) Virus isolations were also performed for 10 different tissue types harvested at Day 1, 2, 3, 5, 6 and 7 post-inoculation.
(TIF)
