# Supplementary figures and images for: Dual microRNA Screens Reveal That the Immune-Responsive miR-181 Promotes Henipavirus Entry and Cell-Cell Fusion
Source: PLoS Pathog. 2016 Oct 26;12(10):e1005974. doi: 10.1371/journal.ppat.1005974 (PMC5082662; doi:10.1371/journal.ppat.1005974)

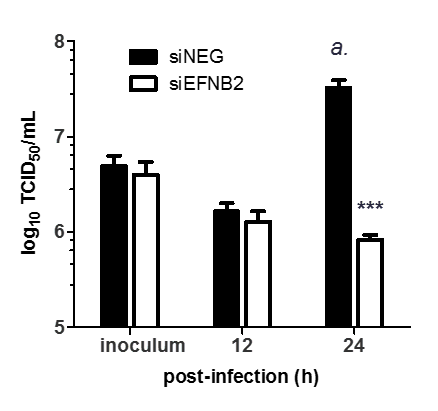

Supplement: S1 Fig — siRNA targeting human EFNB2 or siNEG were transfected into HeLa cells using Dharmafect. 72 hrs after transfection, cells were infected with HeV (MOI 5). At 0, 12 and 24 h.p.i., cell supernatant were harvested and TCID50 analysis were performed. a: p≤0.01 compared to inoculum, ***: p≤0.001 compared to 24 h.p.i. siNEG. (TIF) [file ppat.1005974.s005.tif]

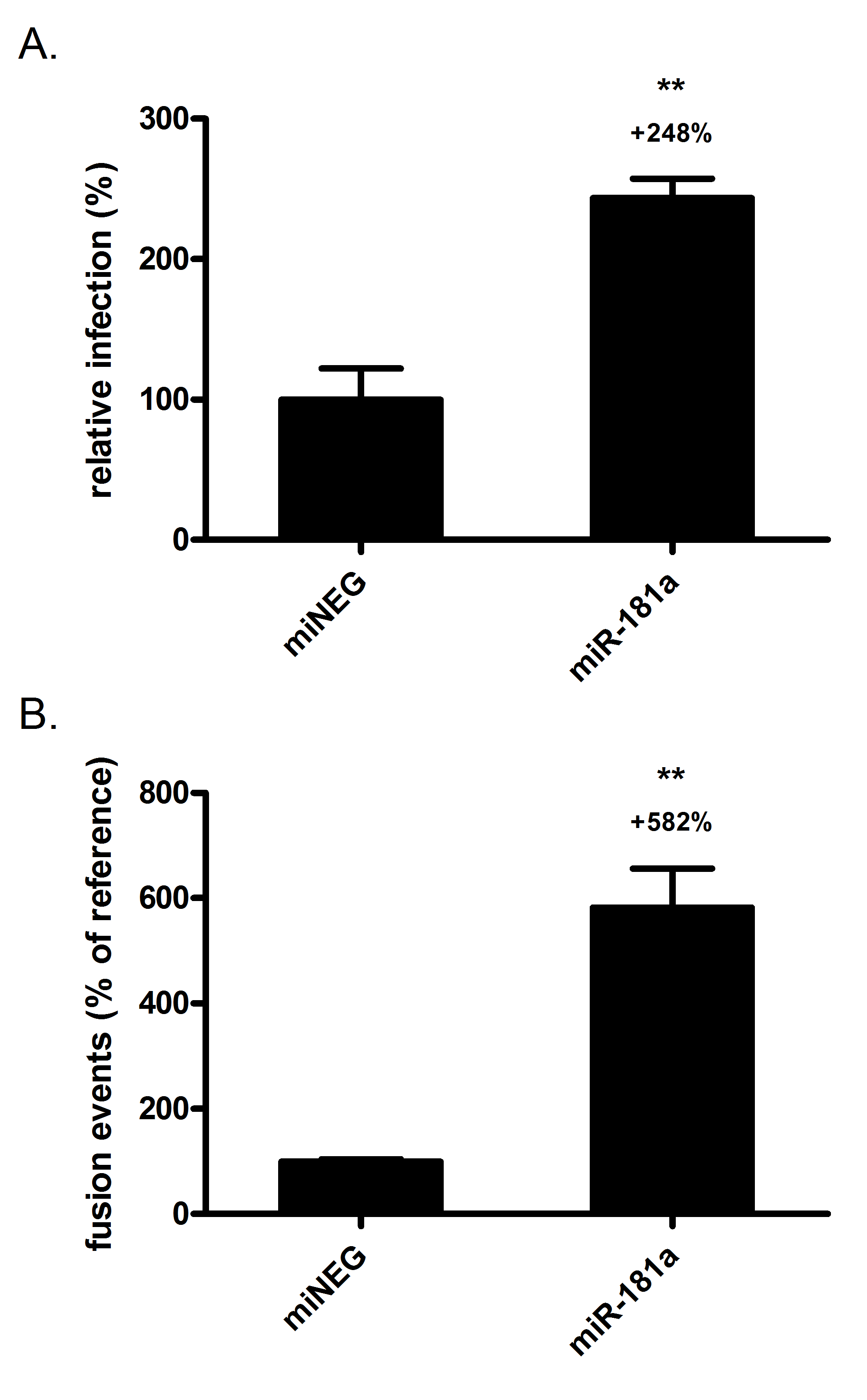

Supplement: S2 Fig — (A) Percentage of HeLa cells stained positive for HeV-P during HeV infection (24 h, MOI 1), 72 h post-transfection with miNEG or miR-181a agonist (25 nM). (B) Relative fusion events in HeLa cells treated with indicated miRNA agonists. (TIF) [file ppat.1005974.s006.tif]

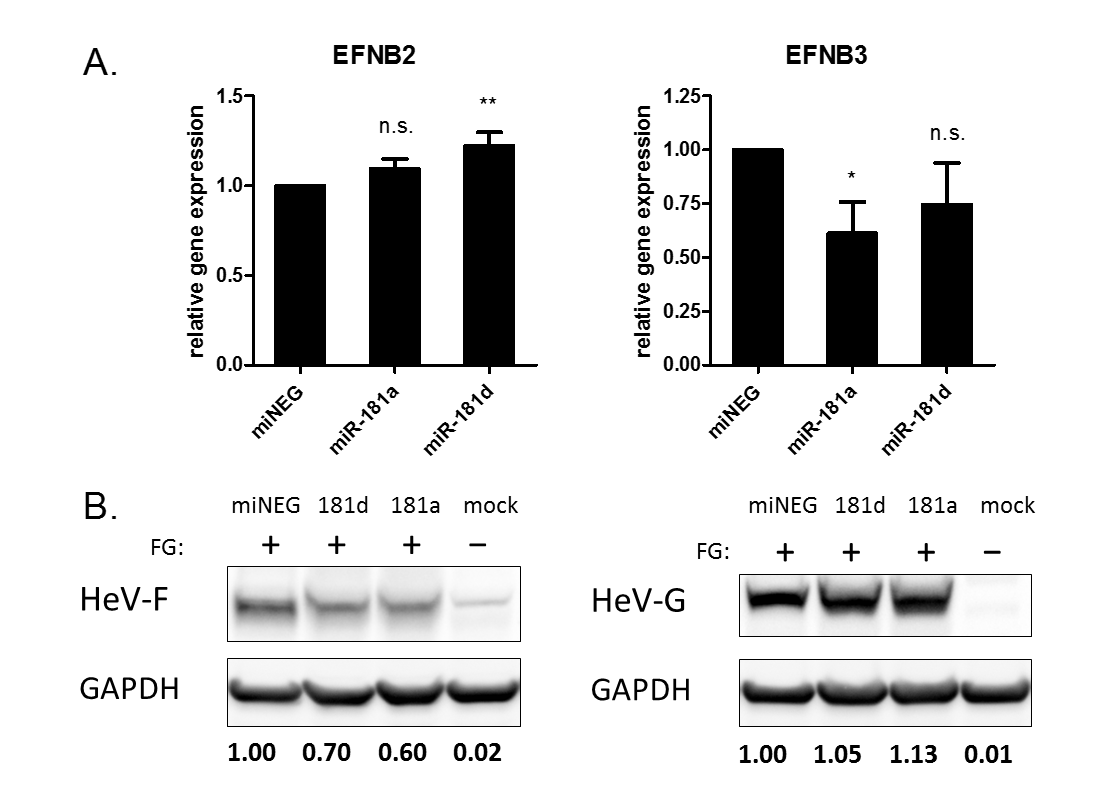

Supplement: S3 Fig — Expression levels of HeV entry receptors ephrin-B2 and–B3 and HeV fusion glycoproteins are minimally affected by mR-181 (A) Relative mRNA levels of EFNB2 and EFNB3 in HeLa cells treated with indicated microRNA agonists (25 nM) for 72 h. n.s. not significant; *p<0.05 compared to miNEG (B) HeV-F and -G protein expression in HeLa cells transfected with cDNA encoding HeV-F and HeV-G (100 ng), in the presence or absence of indicated microRNA agonists (24 h transfection, 25 nM). Quantification of HeV-F and–G is shown numerically relative to GAPDH protein expression levels. (TIF) [file ppat.1005974.s007.tif]

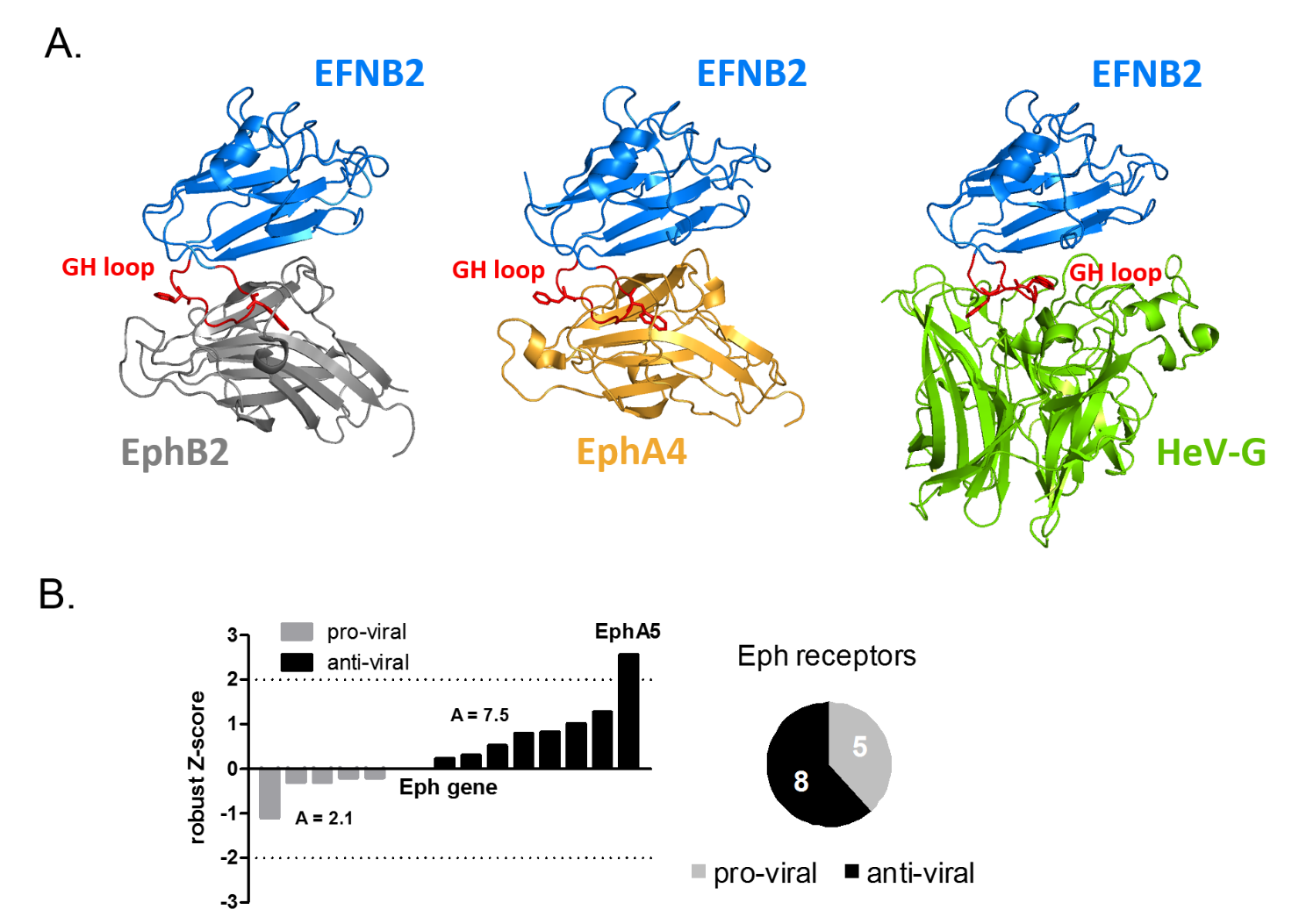

Supplement: S4 Fig — (A) Solved co-crystal structures of ephrin-B2 (blue) in complex with its cellular (EphB2, EphA4) and viral (HeV-G) binding partners indicate that the partners interact with ephrin-B2 primarily via the same binding site on ephrin-B2, the GH loop (red). EphB2 [68] is shown in grey, EphA4 [41] in orange, and viral G-glycoprotein [34] in green. (B) Robust Z scores for all Eph receptors tested in our recently published genome-wide siRNA screen [14]. (TIF) [file ppat.1005974.s008.tif]

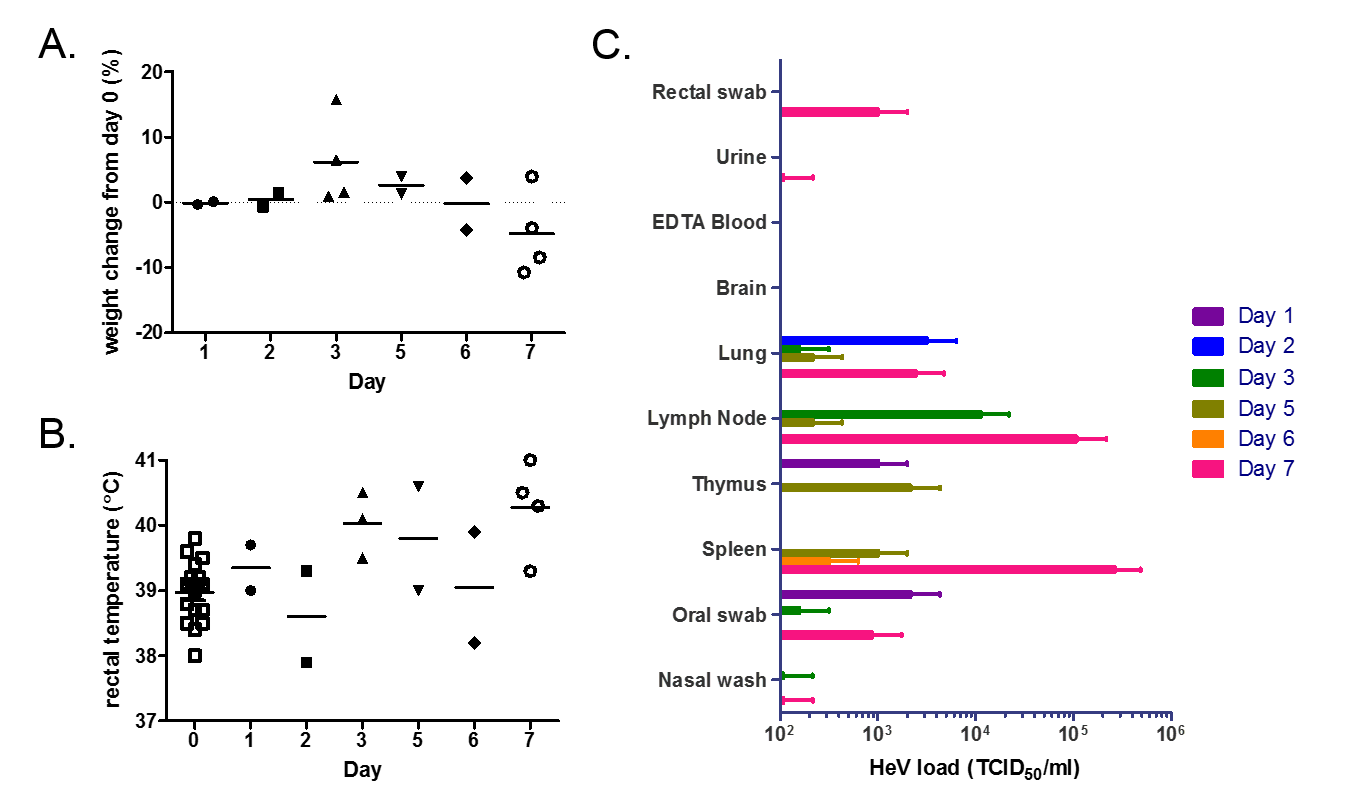

Supplement: S5 Fig — The weight (A) and rectal temperatures (B) of the ferrets were recorded daily through the HeV infection trial. (C) Virus isolations were also performed for 10 different tissue types harvested at Day 1, 2, 3, 5, 6 and 7 post-inoculation. (TIF) [file ppat.1005974.s009.tif]
